# Light energy transduction in liposome-based artificial cells

**DOI:** 10.3389/fbioe.2023.1161730

**Published:** 2023-03-29

**Authors:** Paola Albanese, Fabio Mavelli, Emiliano Altamura

**Affiliations:** ^1^ Department of Earth, Environmental and Physical Sciences, University of Siena, Siena, Italy; ^2^ Department of Biotechnology, Chemistry and Pharmaceutical Sciences, University of Siena, Siena, Italy; ^3^ Department of Chemistry, University of Bari, Bari, Italy

**Keywords:** artificial cell, multi-compartment system, membrane proteins, light transduction, artificial photosyhthesis, photosynthetic proteins, giant vesicle (GV), liposomes

## Abstract

In this work we review the latest strategies for the bottom-up assembly of energetically autonomous artificial cells capable of transducing light energy into chemical energy and support internalized metabolic pathways. Such entities are built by taking inspiration from the photosynthetic machineries found in nature which are purified and reconstituted directly in the membrane of artificial compartments or encapsulated in form of organelle-like structures. Specifically, we report and discuss recent examples based on liposome-technology and multi-compartment (nested) architectures pointing out the importance of this matter for the artificial cell synthesis research field and some limitations and perspectives of the bottom-up approach.

## Introduction

The goal of building cells from scratch has got increasing attention in Synthetic Biology (Stano, 2022) as confirmed by the growing number of publications and scientific networks related to this topic (Schwille et al., 2018; Frischmon et al., 2021; Staufer et al., 2021). Several simplified cell models which exhibit or mimic some characteristics of natural cells, have been successfully developed (Rampioni et al., 2014; Cho and Lu, 2020; Gaut and Adamala, 2021; Ivanov et al., 2021). These cell prototypes, named artificial cells or protocells, are largely employed in the study of fundamental cellular functions (Noireaux and Libchaber, 2004; Herianto et al., 2022) or in biotechnological and health applications (Toparlak and Mansy, 2019; Lussier et al., 2021; Albanese et al., 2022; Sato et al., 2022) since they are suitable for testing new functionalities avoiding the cellular complexity (Jeong et al., 2020). Among the current challenges, the obtainment of energetic autonomy in artificial cells is one of the most pursued (Otrin et al., 2019; Shin, 2019).

From the energetic point of view, living cells can be conceived as compartmentalized microreactors maintained far from equilibrium by a continuous flux of energy in form of high energy compounds (nutrients) or electromagnetic radiation energy (light) (Schrödinger, 1945). Depending on the metabolic capability, organisms can be identified as phototrophs, which use sunlight, and chemotrophs, which gain energy from the oxidation of chemical substances. Phototroph and chemotroph organisms can be further classified into autotrophs, that synthesize complex organic compounds (such as carbohydrates, fats, and proteins) directly from CO_2_, and heterotrophs, which instead necessitate preformed organic nutrients produced by other living organisms.

Actually, the main source of energy for almost all of life on Earth is the Sun. Photosynthetic organisms have evolved a set of protein complexes which perform the transduction of light energy into chemical energy, mainly in form of ATP molecules (light phase). The ATP is in turn required to sustain the anabolic metabolism and the synthesis of organic compounds (dark phase) which nourish heterotrophic species as well (Whitmarsh et al., 1999).

In this paper the attention will be focused on liposome-based artificial cells that mimic the light phase of photosynthesis by transducing light energy into chemical energy in form of a transmembrane pH gradient and eventually, of energy-rich molecules (ATP).

By embedding the photosynthetic machinery of model organisms into synthetic lipid-based compartments, the ultimate goal is to confer energetic autonomy to such entities. First, a brief description of bacterial and plant photosynthetic apparats will be provided. Subsequently, the main examples of proteoliposomes and protocells capable of light energy transduction will be reported, by distinguishing single-compartment and multi-compartment architectures. To conclude, perspectives and limitations of these approaches will be discussed.

## The photosynthetic apparatus in living organisms

In nature, photosynthetic apparats have evolved with large variability and diverse level of complexity between organisms of the different domains of life: Archaea, Bacteria, and Eukarya.

### Photosynthesis in archaea and marine bacteria

The archaeal domain of life encloses microorganisms with extraordinary diverse and even unique metabolic capabilities including organotrophic, lithotrophic and phototrophic pathways evolved to survive harsh environmental conditions (Schäfer et al., 1999).

Phototrophic growth appears to be an exclusive prerogative found in halophilic archaea. Usually, they are aerobic chemoheterotrophs consuming preformed organic substances (mainly amino acids) as source of energy and carbon. However, due to low O_2_ solubility in brine ponds, species like *Halobacterium salinarium*, use sunlight as supplementary energy fuel (Hartmann et al., 1980; Schäfer et al., 1999; Falb et al., 2008).

**FIGURE 1:**
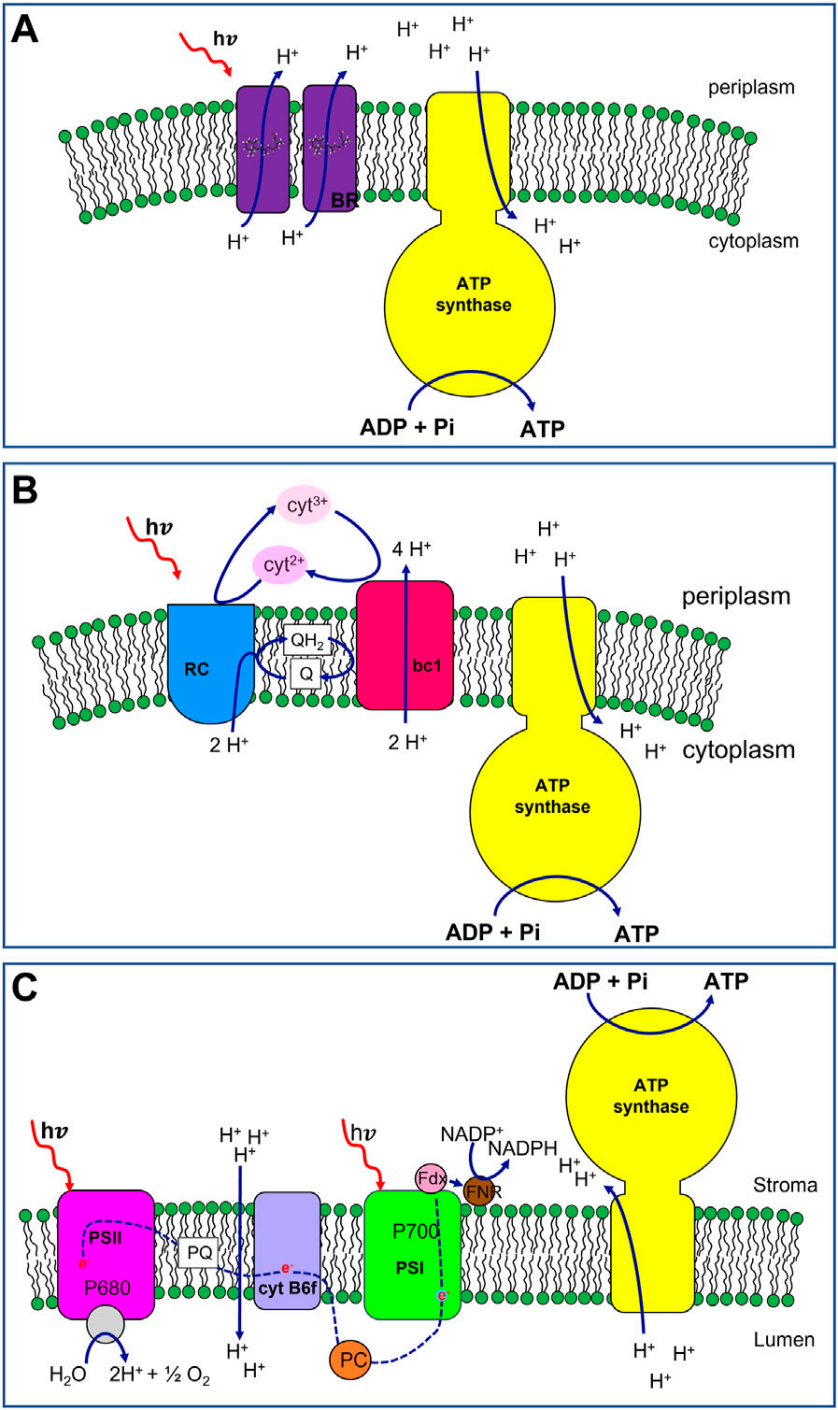
Schematic representation of photosynthetic apparats with increasing complexity belonging to Archea, photosynthetic bacteria and plant cells. **(A)** *Halobacterium salinarium* photophosphorylation machinery. Bacteriorhodopsin (BR, in purple) acts as light-driven proton pump. Upon light excitation, the chromophore, i.e., a retinal group embedded in each protein monomer, undergoes *trans*-to-*cis* photo-isomerization inducing a conformational change in the protein backbone. The all-*trans* original state is regained thanks to the outward movement of protons across the microbial membrane generating a proton potential. The pH gradient is used in the end to drive ATP synthesis by ATP synthase (in yellow). **(B)** Bacterial photophosphorylation machinery from *Rhodobacter sphaeroides*. The primary components of the apparatus are: Reaction Center (RC, in blue), ubiquinol: cytochrome c oxidoreductase (bc1, in red), ATP synthase (in yellow). The bacterial photosynthesis starts with Light Harvesting Complexes (not shown) that absorb incident light and transfer the energy to the Reaction Center. RC catalyses together with bc1, a cyclic redox reaction involving the two couples of electron shuttles: the periplasmic soluble protein cytochrome c2 in the reduced (cyt^2+^) and oxidised (cyt^3+^) form (in pink and light pink, respectively), and liposoluble quinone (Q)/quinol (QH_2_) molecules (in white). During the photo-redox cycle, protons are translocated from the cytoplasm to the periplasm generating a pH gradient across the membrane. This proton-motive force is then exploited by the ATP synthase to synthetize ATP. **(C)** Plant chloroplast photophosphorylation machinery. The main components are 4 membrane protein complexes (i) photosystem II (PSII, in magenta), (ii) cytochrome *b*_*6*_ *f* (in violet), (iii) photosystem I (PSI, in green), and (iv) ATP synthase (in yellow). Upon light absorption, PSII transfers one electron of the special pair of chlorophylls P680 to a plastoquinone molecule (PQ, in white) of the membrane pool. The water splitting, on the lumen side of the membrane, drives the electron for the reduction of the photo-oxidized P680. With the transfer in tandem of two electrons, PQ is reduced to plastoquinol (PQH_2_, not shown) with the uptake of protons from the stroma. PQH_2_ delivers electrons to cytochrome *b*_*6*_ *f*. The *b*_*6*_ *f* oxidizes the plastoquinol molecule transferring electrons to the soluble electron shuttle protein plastocyanin (PC, in orange) while pumping protons across the thylakoid membranes. PC delivers the electrons, one per time, to the PSI. The latter uses light energy and the electron from PC to reduce ferredoxin (Fdx, in pink), an iron-sulfur protein associated with the photosystem from the stromatic side of the membrane. In the end, the electrons of the Fdx are passed to NADP^+^ thanks to the Ferredoxin-NADP^+^ oxidoreductase (FNR, in brown), synthesizing NADPH molecules. The pH gradient, accumulated during the electron transfer (blue dashed line), is the proton motive force driving the synthesis of ATP molecules in the stroma, catalysed by the ATP synthase.

The photophosphorylation mechanism of this archaeon differs from others, relying on a single pigment-protein called bacteriorhodopsin (BR) (Figure 1A, in purple). BR is the smallest light-dependent proton pump known in nature and each protein monomer engages one retinal chromophore as light-harvesting group (a derivative of beta-carotene) (Henderson et al., 1990; Kimura et al., 1997). In the bacterial membrane BR is clustered into hexamers forming two-dimensional hexagonal patches of purple colour due to the presence of the retinal chromophores (Blaurock and Stoeckenius, 1971; Henderson and Unwin, 1975; Essen et al., 1998). Upon light excitation, the retinal group undergoes a conformational change. The original state is regained thanks to the outward movement of protons across the microbial membrane thus generating a proton potential. The pH gradient is used in the end to drive ATP synthesis by ATP synthase (Figure 1A, in yellow) (Lozier et al., 1975).

This photochemical energy conversion mechanism is considered unrelated to other forms of photosynthesis for two main reasons: (i) Halobacteria photochemistry does not require chlorophylls being instead carotenoid-based (ii) redox reactions are not involved in the photocycle since a direct ion transfer occurs upon interaction between light and the pigment-protein complex.

More recently, proteorhodopsin (PR), a retinal-containing integral membrane protein, functioning as a light-driven proton pump as well, was discovered in marine bacteria sharing high amino acid sequence similarity with archaeal BRs (Béjà et al., 2000; Béjà et al., 2001).

### Photosynthetic anoxygenic bacteria: *Rhodobacter sphaeroides*

*R. sphaeroides* 2.4.1 is a Gram-negative, rod-shaped, purple non-sulphur bacterium with a broad range of metabolic capabilities (Woese et al., 1984; Ferguson et al., 1987; Woese, 1987). This organism populates habitats with low-light illumination, namely, ≤10% of full sunlight (Woronowicz and Niederman, 2010; Blankenship, 2014) and experiences the transition between chemotrophic to phototropic growth in response to decreasing of oxygen tension (O_2_ ≤ 3%) (Altamura et al., 2018a). Its photosynthetic apparatus has served as a model for anoxygenic photosynthesis and has been extensively studied over the last 60 years (Sener et al., 2010; Cartron et al., 2014; Sener et al., 2016; Hitchcock et al., 2017). The primary components are, in order of energy utilization: light-harvesting complexes I (LH1) and II (LH2), Reaction Center (RC), ubiquinol: cytochrome c—oxidoreductase (bc1), ATP synthase (ATPsyn). Bacterial photosynthesis starts with Light Harvesting Complexes that absorb incident light and transfer the energy to the Reaction Center (Figure 1B, in blue). RC catalyses together with cytochrome bc1 (Figure 1B, in red), a cyclic redox reaction involving two couples of electron shuttles: periplasmic cytochrome c2 in its reduced (cyt^2+^) and oxidised (cyt^3+^) form (Figure 1B, pink and light pink, respectively), and quinone/quinol (Q/QH_2_) molecules from the membrane pool (Figure 1B, in white). During the photo-redox cycle, protons are translocated from the cytoplasm to the periplasm according to the following stoichiometry:

$${QH}_{2}+2H_{in}^{+}+2{cyt}^{3+}\overset{2h\upsilon }{\to }Q+4H_{out}^{+}+2{cyt}^{2+}$$

where *in* and *out* indicate the cytoplasmic and the periplasmic side of the membrane, respectively. A pH gradient is formed across the membrane and this proton-motive force is then exploited by the ATPsyn (Figure 1B, in yellow) to sustain the phosphorylation of ADP molecules into ATP.

### Chloroplasts and the photophosphorylation apparatus

In photoautotroph eukaryotes, the photosynthetic process occurs in specialized intracytoplasmic organelles named chloroplasts. In addition to the outer double-membrane layer (envelope), they also have an inner system of interconnected membrane-surrounded sacs, called thylakoids, where the photophosphorylation machinery is localised. The organelle aqueous lumen is named stroma and houses the enzymes for metabolic pathways occurring in the organelle most notably starch metabolism and CO_2_-fixing Calvin-Benson cycle for photosynthetic carbon assimilation (Staehelin and Paolillo, 2020).

The main components of the photophosphorylation system are 4 multiprotein complexes (i) photosystem II (PSII, Figure 1C, in magenta), (ii) cytochrome *b*_6_ *f* (Figure 1C, in violet), (iii) photosystem I (PSI, Figure 1C, in green), and (iv) ATP synthase (Figure 1C, in yellow). Light-Harvesting Complexes are also associated with both photosystems, enhancing the light-absorption capabilities of their reaction centres (P680 and P700 for PSII and PSI, respectively).

All these complexes act synergically to generate a light-powered electron flux from a high-redox potential couple (H_2_O/O_2_ E_m_ = 800 mV) to a low-redox potential couple (NAD(P)H/NAD(P)^+^, E_m_ = −300 mV) (Figure 1C, red dashed line). The process is associated with the generation of a transmembrane proton gradient, driving the photophosphorylation of ADP in ATP molecules. The produced energy-rich compounds, NADPH and ATP, are in turn used to sustain the CO_2_-fixing process.

Upon light absorption PSII transfers one electron of the special pair of chlorophylls P680 to a plastoquinone molecule (PQ, Figure 1C, in white) of the membrane pool. The water splitting, catalysed in the proximity of the lumen, drives the electron to the photo-oxidized P680. Once two electrons have been transferred in tandem, PQ is reduced to plastoquinol (PQH_2_) with the uptake of protons from the stroma. PQH_2_ delivers electrons to cytochrome *b*_6_ *f* that oxidizes plastoquinol and uses the gained electrons to reduce the electron-shuttle protein plastocyanin (PC, Figure 1C, in orange) contextually pumping protons across the thylakoid membranes. PC delivers the electrons to the photo-oxidized PSI which preliminarily has reduced ferredoxin (Fdx, Figure 1C, in pink), an iron-sulfur protein associated with the photosystem from the stromatic side of the membrane. Finally, the electrons of the Fdx are passed to NADP^+^ by the Ferredoxin-NADP^+^ oxidoreductase (FNR, Figure 1C, in brown), synthesizing NADPH molecules. The pH gradient, accumulated during the electron transfer pathway, is the proton motive force driving the synthesis of ATP molecules in the stroma. In *Spinacia oleracea* chloroplasts, a complete rotational catalysis of ATPsyn requires 14 H^+^ and brings to the production of 3 ATP molecules (Hahn et al., 2018).

Considering that a proton gradient of approximately 12 H^+^ is produced for every O_2_ molecule evolved (Furbank and Badger, 1983), the overall stoichiometric equation of the process is:

$$8\;photons+2H_{2}O+2\;{NADP}^{+}+2.57\;ADP+2.57\;P_{i}\to O_{2}+2\;NADPH+2.57\;ATP$$

and describes the unidirectional electron transfer from H_2_O to NADP^+^. The production of energy-rich molecules must be strictly balanced by their consumption to avoid an over-production of highly reactive chemical species leading to photodamage (Kramer and Evans, 2011).

## Light transduction in artificial compartmentalized systems

This section presents and discusses examples of artificial compartments transducing light into chemical energy. The attention will be focused mainly on lipid-based nanometric or micrometric compartments where the formation of a transmembrane pH gradient is the first step like in the natural light conversion.

### Proteoliposomes: single compartment strategy

Starting from the end of the last century, transmembrane proteins have been extensively studied by reconstitution into nano-sized lipid vesicles as simplified membrane models, obtaining proteoliposomes (Rigaud et al., 1995). This allowed to explore the characteristics of the proteins avoiding the complexity of the native membrane and the interference with other cellular components or side reactions. Particular attention was paid to energy-transducing membrane proteins, such as BR and FOF1-ATP synthases. When BR is reconstituted alone into phospholipid vesicles is capable of acidifying the liposome internal milieu upon illumination (van Dijck and van Dam, 1982). The case of the co-reconstitution of BR hexamers from *Halobacterium halobium* and FOF1-ATPsyn from bovine mitochondria in liposome is the first example of light energy transducer lipid compartments reported in 1974 by Racker and Stoeckenius (Racker and Stoeckenius, 1974). It was proven that these proteoliposomes could photo-synthesize ATP from ADP and inorganic phosphate under light irradiation. Although the observed rate of ATP synthesis was only 0.1% of the oxidative phosphorylation rate found in mitochondria, proteoliposomes embedding BR and ATP synthase became an ideal model to study the mechanism of energy coupling. A subsequent work pointed out that ATP synthesis yield is influenced by the protein purification method, the co-reconstitution procedure, and the type of phospholipids used which affects the protein distribution among liposomes (Van Der Bend et al., 1984).

Indeed, bacteriorhodopsin in monomeric form performs much better than in form of purple membrane patches being more homogeneously distributed among liposomes. This results in a 6.2 times higher ATP production rate (Wagner et al., 1987).

In the following years, several authors reported the possibility to reconstitute FOF1-ATP synthase complex along with monomeric bacteriorhodopsin in proteoliposomes, as well. A comparison among examples of these semi-synthetic photoactive organelles is reported in Otrin et al., 2019, where the type of compartment and the light transducing efficiency are also listed.

Later in 1997, Moore and co-workers replaced BR with an artificial reaction centre prepared by linking a synthetic tetraarylporphyrin to both a naphthoquinone moiety and a norbornene system with a carboxylic group and a carotenoid polyene (Steinberg-Yfrach et al., 1997), subsequently coupled with ATPsyn in an artificial photosynthetic membrane (Steinberg-Yfrach et al., 1998).

More recently, mimicking the bacterial photosynthetic apparatus, the Rection Center extracted from the R26 mutant of *R. sphaeroides* has been reconstituted in the GUV membrane with the 90% of physiological orientation. These giant proteoliposomes generate a transmembrane proton gradient under continuous light irradiation by increasing the internal pH with a rate of 0.061 ± 0.004 pH units per minute and exhibit a retained photo-efficiency of 80% after 24 h (Altamura et al., 2017a).

Although the co-reconstitution of bc1 along with RC in the GUV membrane with a correct orientation is still a challenging task (Hardy et al., 2018), it has been also shown that the bacterial RC proteins can be coupled with the bc1 extracted from bovine heart mitochondria to obtain a more efficient hybrid system in comparison to the bacterial one (Altamura et al., 2017b; Altamura et al., 2021a). This, in principle, gives the chance to improve the model bacterial apparatus with the most efficient protein complexes extracted from mammalian cells.

### Multi-compartmentalized giant vesicles

Different classes of multi-compartment systems have been developed and proposed to mimic the architecture of living cells including liposomes within layer-by-layer capsules (capsosomes), liposome-in-liposome (vesosomes), polymersome-in-polymersome architectures and multisomes (Schoonen and van Hest, 2016). Herein we focus the attention exclusively on multi-compartment architecture based on giant lipid vesicles.

**FIGURE 2:**
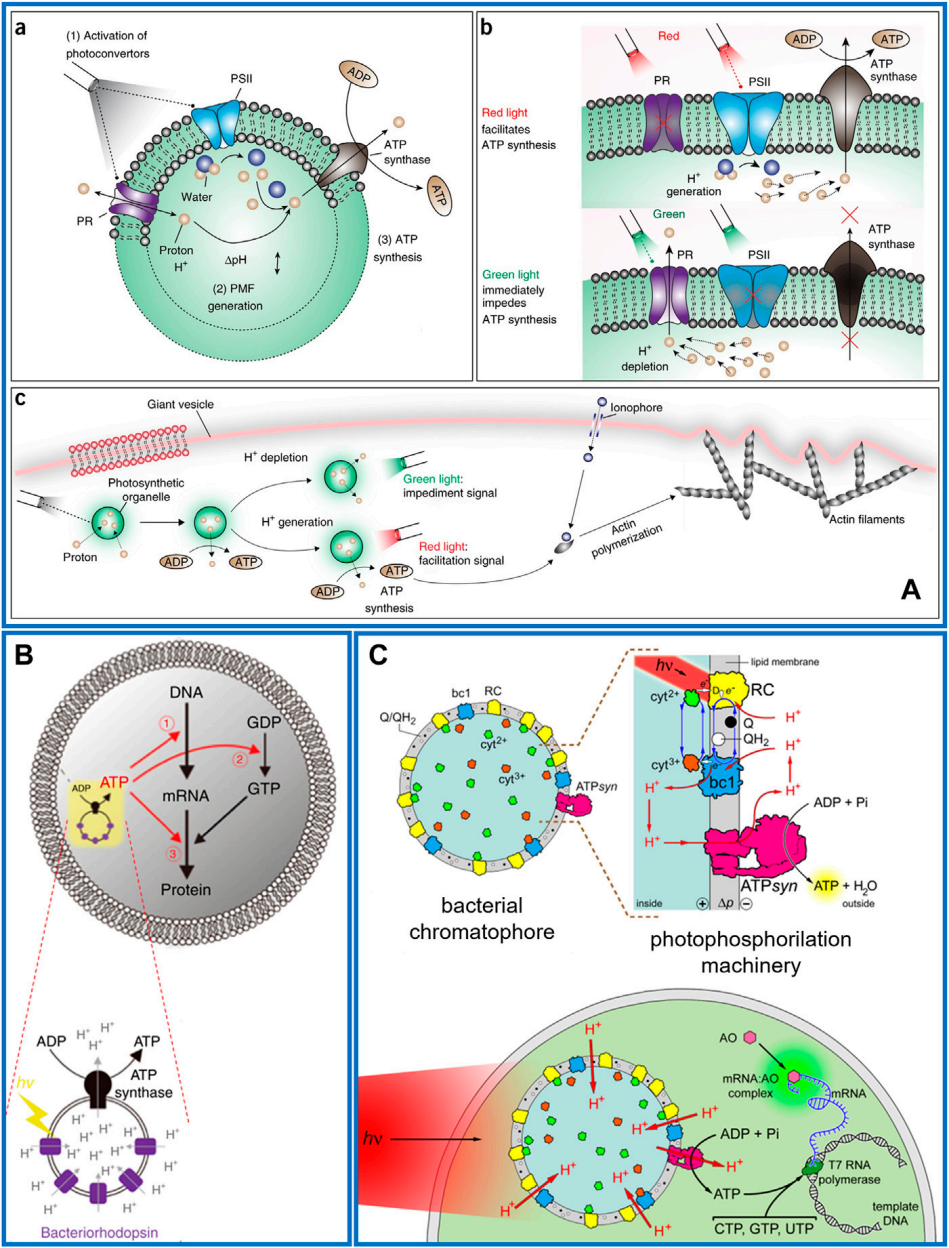
Photosynthetic multicompartment artificial cells. **(A)** a) Nanometric lipid vesicle with two complementary photoconverters (PSII and PR) and an ATP synthase reconstituted into the membrane. (b) Red light illumination triggers PSII that generates a proton gradient across the lipid membrane then exploited by ATP synthase for ATP synthesis. Green light activates PR causing proton depletion and impeding ATP synthesis. (c) Photosynthetic liposomes used as organelle energy modules when encapsulated in a giant vesicle, for actin polymerization and morphological change of the vesicle after optical stimulation (Lee et al., 2018). **(B)** Schematic representation of a giant lipid vesicles encapsulating nanometric liposome as artificial photosynthetic organelle, which consists of BR and ATP synthase. The ATP photo-produced can sustain mRNA synthesis (➀), guanosine diphosphate (GDP) phosphorylation (➁) or tRNA aminoacylation (➂) (Berhanu et al., 2019). **(C)** Chromatophores extracted from *R. sphaeroides* are encapsulated inside giant phospholipid vesicles made of POPC and illuminated to generate a proton motive force (Δp ∼ 130 mV) across the membrane. Co-encapsulated ADP, Pi, GTP, CTP, UTP, T7 RNA polymerase (dark green), and a DNA template give rise to an out-of-equilibrium system of coupled reactions: namely, photophosphorylation and DNA transcription. The biosynthesized mRNA is revealed by acridine orange (AO, pink), which binds it, forming a green-fluorescent complex (Altamura et al., 2021b).

In 2018, the first example was realized with engineered switchable photosynthetic organelles (100 nm diameter) encapsulated in giant lipid vesicles (10–100 µm diameter) (Figure 2A). The synthetic organelles were acting as energy modules enabling the conversion of light energy into ATP molecules, in turn used to sustain an internalized anabolic reaction. In the nano-liposome membrane two complementary photoconverters were co-reconstituted: plant-derived photosystem II (PSII) and bacterial-derived proteorhodopsin (PR) along with ATPsyn. Upon white light illumination, the PSII and PR generated a proton motive force across the proteoliposome membrane that was sufficient to induce ATP synthesis outside the artificial organelles (Figure 2A) (Lee et al., 2018). The maximum turnover number of the ATP synthase was 4.3 ± 0.1 s^−1^. The artificial organelles sustained ATP conversion for 3 days (half-maximum efficiency) at room temperature and for 1 month at 4°C. Optical independent activation of the two photoconverters allowed dynamic control of ATP synthesis since red light facilitates and green light impedes ATP synthesis (Figure 2A). This artificial system was able to sustain two ATP-dependent reactions powered by light, such as carbon fixation and actin polymerization, altering, with the latter, the vesicle morphology (Figure 2A). In the can be used to develop biomimetic vesicle systems with regulatory networks that exhibit homeostasis and complex cellular behaviours.

One year later, Berhanu and co-workers reported the co-encapsulation in GUVs of a cell-free protein synthesis system and proteoliposomes containing BR and ATP synthase. The photoproduced ATP sustained the synthesis of BR or constituent proteins of ATP synthase, that spontaneously integrated into the artificial photosynthetic organelles enhancing its photosynthetic activity (Figure 2B). The ATPsyn turnover number in the first 5 min of proteoliposome bulk activity resulted in 8.3 ± 0.3 s^−1^, while when encapsulated in GUVs it reduces to one-third (Berhanu et al., 2019).

More recently, it has been shown that the entire photosynthetic apparatus of a living organism can be encapsulated into GUVs in form of chromatophores, natural nanometric proteoliposomes extracted from *R. sphaeroides*. Under specific growth conditions this bacterium forms cytoplasmic membrane invaginations in which the photosynthetic system is confined (Feniouk et al., 2002; Noble et al., 2018). After a single French press step, bacterial cells are broken inducing the spontaneous closure of the present invaginations into chromatophores. The nano-vesicles still trap the intact photosynthetic apparat in their membranes with the physiological orientation of all protein complexes. These organelles were encapsulated in GUVs along with a commercial kit for DNA transcription showing that the photo-produced ATP was sufficient to sustain the synthesis of mRNA molecules (Figure 2C). The estimated turnover of the ATPsyn was around 80 s^−1^ (Altamura et al., 2021b).

## Discussion and perspectives

The strategies explored for the implementation of light-energy transducing artificial cells based on liposome technology, rely either on single- and multi-compartment designs.

The single-compartment approach consists in the incorporation of purified photo-transducing proteins in the membrane bilayer of synthetic liposomes with the desired orientation. Examples of such entities obtained with a single protein type include the reconstitution of bacteriorhodopsin in hexameric (membrane patches) (Racker and Stoeckenius, 1974) or monomeric form (Wagner et al., 1987); the Reaction Center from *R. sphaeroides* (Altamura et al., 2017a) and even an artificial transmembrane proton pump based on a carotene-porphyrin-naphthoquinone molecular triad (Steinberg-Yfrach et al., 1997). In all cases, upon light activation a pH gradient, acidic inside, could be detected.

Certainly, to assemble a proper photosynthetic machinery the light-activated proton-pump must be coupled with the phosphorylating complex ATP synthase. In this framework, many research groups managed to co-reconstitute the BR-ATPsyn protein-couple into nanometrical liposomes. This architecture was firstly achieved in the seminal work, Racker and Stoeckenius (Racker and Stoeckenius, 1974) where such nano-sized proteoliposomes were assembled and implemented as light energy transducers. The system mimicked the photosynthetic machinery of halobacteria which still represents the simplest apparatus capable of trapping and converting light energy in a photochemical gradient, in turn used to sustain ADP phosphorylation.

Subsequent works showed an improved overall efficiency of this system by (i) using monomeric BR form (Wagner et al., 1987) (ii) coupling BR with ATP synthases extracted from different organisms (Richard and Graber, 1992; Pitard et al., 1996) (iii) appropriately designing the lipid membrane, and (iv) optimizing the co-reconstitution of the protein complexes (Otrin et al., 2019). In the case of the enzymatic apparatus of the bacterium *R. sphaeroides*, a third protein complex, beside RC and ATPsyn, should be considered: the cytochrome bc1. The integration of bc1 in the RC-ATPsyn system would allow potentially unlimited recycling of the reaction redox cofactors boosting the efficiency of the system (Altamura et al., 2017a, 2021a).

Moving forward, according to the multi-compartment design (Altamura et al., 2021c), light-transducing proteoliposomes have been envisaged as photosynthetic organelle-like modules in artificial cell construction. Once encapsulated in GUVs, they efficiently photo-support several internalized metabolic reaction networks (Lee et al., 2018; Berhanu et al., 2019). An alternative hybrid multi-compartment strategy was pursued by encapsulating in the GUV lumen chromatophores, i.e., easy-to-extract and ready-to-use nano-sized photosynthetic lipid compartments directly extracted from bacterial cells (Altamura et al., 2021b).

Table 1 shows a comparison between the most relevant systems described in this review summarizing the main characteristics of the different lipid architectures.

**TABLE 1:** Comparison of different photosynthetic lipid architectures for light-induced transmembrane pH gradient generation and ATP synthesis.

Proteoliposome in aqueous phase	Transmembrane proton motive force					
	Proton Pump	ATPsyn Source	Lipid compartments	Energy	Efficiency	Ref
	BR (patches) *Halobacterium halobium*	None	Soybean phospholipid SUVs	200 mW/cm^2^	50–200 nmoles of H^+^/mg BR	Racker and Stoeckenius, (1974)
	BR (monomer) *Halobacterium halobium*	None	Soybean lecithin SUVs	400 mW/cm^2^	108 nmol H^+^/min/BR	Wagner et al. (1987)
	Synthetic carotene– porphyrin–naphthoq uinone Molecular Triad	None	PS:DOPC 2:3 M ratio SUVs	5 mW/cm^2^	0.3 nM H^+^/min/BR	Steinberg-Yfrach et al. (1997)
	RC *Rhodobacter sphaeroides*	None	POPC GUVs	200 mW/cm^2^	200 nmol H^+^/min/RC	Altamura et al. (2017a)

	Light-driven ADP phosphorilation					
	Proton Pump	ATPsyn Source	Proteoliposomes	Energy	Efficiency	Ref
	BR *Halobacterium halobium*	Bovine heart mitochondria	Soybean phospholipids SUVs	200 mW/cm^2^	312 nmol Glucose‐6‐ P/min/mg enzyme	Racker and Stoeckenius, (1974)
	BR *Halobacterium salinarium*	*Rhodospirillum rubrum*	Soybean lecithin SUVs	400 mW/cm^2^	280 nmol ATP/min/mg enzyme	Wagner et al. (1987)
	Synthetic carotene– porphyrin–naphthoq uinone Molecular Triad	Spinach chloroplasts	PC:PA 10:1, 20 mol% chol SUVs	0.1 mW/cm^2^	7 ATP/s per enzyme	Steinberg-Yfrach et al. (1998)
	BR *Halobacterium salinarium*	Spinach chloroplasts	PC/PA mixture SUVs	150 W (white light)	204 nmol ATP/min/mg enzyme	Richard and Graber, (1992)
	BR *Halobacterium salinarium*	*Bacillus* PS3	PC:PA 9:1 SUVs	800 W (*λ* = 500–650 nm)	7 ATP/s per enzyme	Pitard et al. (1996)
	PSII from Spinach and BR from Gamma proteobacteriumID: AAG10475	*Bacillus pseudofirmus*	DOPC:POPE:DOPS:Chol 2:1:2:1, 1 mol% PEG2000PE SUVs	1.14 mW/cm^2^ (*λ* = 660 nm)	4.3 ATP/s per enzyme	Lee et al. (2018)
	BR *Halobacterium salinarum* R1	*Bacillus* PS3	Soybean PC, 30 mol% chol SUVs	10 W/cm^2^ (*λ* = 500 nm)	8.3 ATP/s per enzyne	Berhanu et al. (2019)
	RC/bc1 *Rhodobacter spheroides*	*Rhodobacter sphaeroides*	Bacterial Chromatophores	25 mW/cm^2^ (*λ* = 860 nm)	80 ATP/s per enzyme	Altamura et al. (2021b)

Multicompartment GUVs		Light‐driven ADP phosphorilation				
	Proton Pump	ATPsyn Source	Organelles in GUVs	Energy	Efficiency	Ref
	PSII from Spinach and BR from Gamma proteobacterium ID: AAG10475	*B. pseudofirmus*	DOPC:POPE:DOPS:Chol 2:1:2:1, 1 mol% PEG2000PE SUVs in POPC:POPE:POPG:Chol 2:1:1:1 GUVs	1.14 mW/cm^2^ (*λ* = 660 nm)	Not explicitly reported	Lee et al. (2018)
	BR *Halobacterium salinarum* R1	*Bacillus* PS3	Soybean PC, 30% Chol SUVs in POPC:Chol:PEG2000PE 5.75:4:0.25 GUVs	10 W/cm^2^ (*λ* = 500 nm)	5 µM ATP/min per GUV	Berhanu et al. (2019)
	RC/bc1 *Rhodobacter sphaeroides*	*R. sphaeroides*	Bacterial Chromatophores in POPC GUVs	25 mW/cm^2^ (*λ* = 860 nm)	1.3 µM ATP/min per GUV	Altamura et al. (2021b)

List of symbols: Chol, cholesterol; PA, phosphatidic acid; PC, phosphatidylcholine; PS, phosphatidylserine; DOPC, Dioleoylphosphatidylcholine; DOPS, 1,2-dioleoyl-sn-glycero-3-phospho-L-serine; POPE, 1-palmitoyl-2-oleoyl phosphatidylethanolamine; POPC, 1-palmitoyl-2-oleoyl-*sn*-glycero-3-phosphocholine; POPG, 1-palmitoyl-2-oleoyl-*sn*-glycero-3-phospho-(1′-rac-glycerol); PEG, Poly (ethylene glycol); GUVs, Giant Unilamellar Vesicles; SUVs, Small Unilamellar Vesicles.

Despite the milestones achieved in recent years, some critical points remain in the fabrication of light-powered artificial cells, and these concern the reliability of the preparation procedures, the photo-stability and robustness of the protocells themselves (Amati et al., 2020).

Traditional preparation methods such as phase transfer, natural swelling, and electro-formation (Walde et al., 2010), produce a widely distributed protocell population in size and solute content (Altamura et al., 2018b) compared to microfluidic approaches (Ai et al., 2020). This would require time-consuming and thorough statistical studies and simulations (Calviello et al., 2013; Shirt-Ediss et al., 2014; D’aguanno et al., 2015; Altamura et al., 2020) to estimate the yield of the production of truly functional liposomes, an analysis that is often lacking in pioneering works.

A similar point also concerns the co-reconstitution in the vesicle lipid bilayer of two or more transmembrane proteins. This is a very challenging task since it requires to find the experimental conditions that maximize the incorporation yield of different enzymes while keeping the desired alignment in the lipid membrane. In addition, too often the confocal microscopy analysis on the giant lipid architectures is limited to a few successful cases without giving exact information on the feasibility of the method used. In most cases, the time window used to monitor photoactivity is limited to the interval strictly necessary to highlight the functionality of the photosynthetic apparatus, using an excess of light radiation to increase system performance. These non-physiological working conditions can induce a rapid degradation of membrane proteins which is not evidenced by long-term photoactivity studies, reported only by a few authors (Altamura et al., 2017a; Lee et al., 2018).

The investigations on the morphological stability of the protocells over time, together with the robustness of the selected photosynthetic apparatus, could provide further insights to define the most suitable preparation procedure and the best photosynthetic apparatus to implement in light transducing protocells.

Alternatively, the use of physiological photosynthetic organelles evidenced a higher ATPsyn turnover number (Altamura et al., 2021b) suggesting that the encapsulation of the enzymes in their natural lipid environment (as whole mitochondria, chloroplasts or thylakoid membranes) would maximise their catalytic activity. A recent example of this approach is reported by Miller and co-workers, who coupled photosynthetic light phase catalysed by thylakoid membranes with a synthetic reaction pathway for CO_2_ fixation in water-in-oil droplets (Miller et al., 2020).

## References

[B1] Ai Y. Xie R. Xiong J. Liang Q. (2020). Microfluidics for biosynthesizing: From droplets and vesicles to artificial cells. Small 16 (9), 1903940. 10.1002/smll.201903940 31603270

[B2] Albanese P. Cataldini S. Ren C. Z.-J. Valletti N. Brunetti J. Chen J. L.-Y. (2022). Light-switchable membrane permeability in giant unilamellar vesicles. Pharmaceutics 14, 2777. 10.3390/pharmaceutics14122777 36559270 PMC9780837

[B3] Altamura E. Albanese P. Marotta R. Milano F. Trotta M. Stano P. (2021b). Chromatophores efficiently promote light-driven ATP synthesis and DNA transcription inside hybrid multi compartment artificial cells. Proc. Natl. Acad. Sci. U. S. A. 118 (7), e2012170118. 10.1073/pnas.2012170118 33526592 PMC7896284

[B4] Altamura E. Albanese P. Mavelli F. Stano P. (2021c). The rise of the nested multicompartment model in synthetic cell research. Front. Mol. Biosci. 8, 750576. 10.3389/fmolb.2021.750576 34540903 PMC8446550

[B5] Altamura E. Albanese P. Milano F. Giotta L. Trotta M. Ferretta A. (2021a). Optimizing enzymatic photo‐redox cycles by a hybrid protein complex chain. ChemPhotoChem 5 (1), 26–31. 10.1002/cptc.202000176

[B6] Altamura E. Albanese P. Stano P. Trotta M. Milano F. Mavelli F. (2020). Charge recombination kinetics of bacterial photosynthetic reaction centres reconstituted in liposomes: Deterministic versus stochastic approach. Data 5, 53. 10.3390/data5020053

[B7] Altamura E. Carrara P. D’Angelo F. Mavelli F. Stano P. (2018b). Extrinsic stochastic factors (solute partition) in gene expression inside lipid vesicles and lipid-stabilized water-in-oil droplets: A review. Synth. Biol. 3, ysy011. 10.1093/synbio/ysy011 PMC7445889 32995519

[B8] Altamura E. Fiorentino R. Milano F. Trotta M. Palazzo G. Stano P. (2017b). First moves towards photoautotrophic synthetic cells: *In vitro* study of photosynthetic reaction centre and cytochrome bc1 complex interactions. J. Biophys. Chem. 229, 46–56. 10.1016/j.bpc.2017.06.011 28688734

[B9] Altamura E. Mavelli F. Milano F. Trotta M. (2018a). Photosynthesis without the organisms: The bacterial chromatophores. Adv. Bionanomaterials - Lect. Notes Bioeng., 165–173.

[B10] Altamura E. Milano F. Tangorra R. R. Trotta M. Omar O. H. Stano P. (2017a). Highly oriented photosynthetic reaction centers generate a proton gradient in synthetic protocells. Proc. Natl. Acad. Sci. U. S. A. 114, 3837–3842. 10.1073/pnas.1617593114 28320948 PMC5393214

[B11] Amati A. M. Graf S. Deutschmann S. Dolder N. von Ballmoos C. (2020). Current problems and future avenues in proteoliposome research. Biochem. Soc. Trans. 48, 1473–1492. 10.1042/bst20190966 32830854

[B12] Béjà O. Aravind L. Koonin E. V. Suzuki M. T. Hadd A. Nguyen L. P. (2000). Bacterial rhodopsin: Evidence for a new type of phototrophy in the sea. Science 289, 1902–1906. 10.1126/science.289.5486.1902 10988064

[B13] Béjà O. Spudich E. N. Spudich J. L. Leclerc M. DeLong E. F. (2001). Proteorhodopsin phototrophy in the ocean. Nature 411, 786–789. 10.1038/35081051 11459054

[B14] Berhanu S. Ueda T. Kuruma Y. (2019). Artificial photosynthetic cell producing energy for protein synthesis. Nat. Commun. 10, 1325. 10.1038/s41467-019-09147-4 30902985 PMC6430821

[B15] Blankenship R. E. (2014). Molecular mechanisms of photosynthesis 2nd Ed. Wiley Blackwell.

[B16] Blaurock A. E. Stoeckenius W. (1971). Structure of the purple membrane. Nat. New Biol. 233, 152–155. 10.1038/newbio233152a0 5286750

[B17] Calviello L. Stano P. Mavelli F. Luisi P. L. Marangoni R. (2013). Quasi-cellular systems: Stochastic simulation analysis at nanoscale range. Bioinformatics 14, S7. 10.1186/1471-2105-14-S7-S7 PMC3633058 23815522

[B18] Cartron M. L. Olsen J. D. Sener M. Jackson P. J. Brindlay A. M. Quian P. (2014). Integration of energy and electron transfer processes in the photosynthetic membrane of *Rhodobacter sphaeroides* . Biochim. Biophys. Acta-Bioenerg. 1837, e118–e1780. 10.1016/j.bbabio.2014.05.314 PMC4143486 24530865

[B19] Cho E. Lu Y. (2020). Compartmentalizing cell-free systems: Toward creating life-like artificial cells and beyond. ACS Synth. Biol. 9, 2881–2901. 10.1021/acssynbio.0c00433 33095011

[B20] D’Aguanno E. Altamura E. Mavelli F. Fahr A. Stano P. Luisi P. L. (2015). Physical routes to primitive cells: An experimental model based on the spontaneous entrapment of enzymes inside Micrometer-Sized liposomes. Life 5, 969–996. 10.3390/life5010969 25793278 PMC4390888

[B21] Essen L. O. Siegert R. Lehmann W. D. Oesterhelt D. (1998). Lipid patches in membrane protein oligomers—Crystal structure of the bacteriorhodopsin-lipid complex. Proc. Natl. Acad. Sci. U. S. A. 95, 11673–11678. 10.1073/pnas.95.20.11673 9751724 PMC21699

[B22] Falb M. Müller K. Königsmaier L. Oberwinkler T. Horn P. von Gronau S. (2008). Metabolism of halophilic archaea. Extremophiles 12, 177–196. 10.1007/s00792-008-0138-x 18278431 PMC2262144

[B23] Feniouk B. A. Cherepanov D. A. Voskoboynikova N. E. Mulkidjanian A. Y. Junge W. (2002). Chromatophore vesicles of *Rhodobacter capsulatus* contain on average one FoF1-ATP synthase each. Biophysical J. 82, 1115–1122. 10.1016/s0006-3495(02)75470-2 PMC1301917 11867431

[B24] Ferguson S. T. Jackson J. B. McEwan A. G. (1987). Anaerobic respiration in the *rhodospirillaceae*: Characterisation of pathways and evaluation of roles in redox balancing during photosynthesis. FEMS Microbiol. Rev. 46, 117–143. 10.1111/j.1574-6968.1987.tb02455.x

[B25] Frischmon C. Sorenson C. Winikoff M. Adamala K. P. (2021). Build-a-Cell: Engineering a synthetic cell community. Life 11, 1176. 10.3390/life11111176 34833052 PMC8618533

[B26] Furbank R. T. Badger M. R. (1983). Oxygen exchange associated with electron transport and photophosphorylation in spinach thylakoids. Biochim. Biophys. Acta 723, 400–409. 10.1016/0005-2728(83)90047-6

[B27] Gaut N. J. Adamala K. P. (2021). Reconstituting natural cell elements in synthetic cells. *Adv. Biol.* (Weinh). 5, e2000188. 10.1002/adbi.202000188 33729692

[B28] Hahn A. Vonck J. Mills D. J. Meier T. Kuhlbrandt W. (2018). Structure, mechanism, and regulation of the chloroplast ATP synthase. Science 360, eaat4318. 10.1126/science.aat4318 29748256 PMC7116070

[B29] Hardy D. Desuzinges Mandon E. Rothnie A. J. Jawhari A. (2018). The yin and yang of solubilization and stabilization for wild-type and full-length membrane protein. Methods 147, 118–125. 10.1016/j.ymeth.2018.02.017 29477816

[B30] Hartmann R. Sickinger H.-D. Oesterhelt D. (1980). Anaerobic growth of halobacteria. Proc. Natl. Acad. Sci. U.S.A. 77, 3821–3825. 10.1073/pnas.77.7.3821 6933439 PMC349718

[B31] Henderson R. Baldwin J. M. Ceska T. A. ZemlinBeckmann E. Downing K. H. (1990). Model for the structure of bacteriorhodopsin based on high-resolution electron cryo-microscopy. J. Mol. Biol. 213, 899–929. 10.1016/s0022-2836(05)80271-2 2359127

[B32] Henderson R. Unwin P. N. T. (1975). Three-dimensional model of purple membrane obtained by electron microscopy. Nature 257, 28–32. 10.1038/257028a0 1161000

[B33] Herianto S. Chien P.-J. Ho J. A. Tu H.-L. (2022). Liposome-based artificial cells: From gene expression to reconstitution of cellular functions and phenotypes. Biomater. Adv. 142, 213156. 10.1016/j.bioadv.2022.213156 36302330

[B34] Hitchcock A. Hunter C. N. Sener M. (2017). Determination of cell doubling times from the return-on-investment time of photosynthetic vesicles based on atomic detail structural models. J. Phys. Chem. B 121 (15), 3787–3797. 10.1021/acs.jpcb.6b12335 28301162 PMC6362981

[B35] Ivanov I. Castellanos S. L. Balasbas S. Otrin L. Marušič N. Vidaković-Koch T. (2021). Bottom-up synthesis of artificial cells: Recent highlights and future challenges. Annu. Rev. Chem. Biomol. Eng. 12, 287–308. 10.1146/annurevchembioeng-092220-085918 34097845

[B36] Jeong S. Nguyen H. T. Kim C. H. Ly M. N. Shin K. (2020). Toward artificial cells: Novel advances in energy conversion and cellular motility. Adv. Funct. Mater. 30, 1907182. 10.1002/adfm.201907182

[B37] Kimura Y. Vassylyev D. G. Miyazawa A. Kidera A. Matsushima K. Murata K. (1997). Surface of bacteriorhodopsin revealed by high-resolution electron crystallography. Nature 389, 206–211. 10.1038/38323 9296502

[B38] Kramer D. M. Evans J. R. (2011). The importance of energy balance in improving photosynthetic productivity. Plant physiol. 155, 70–78. 10.1104/pp.110.166652 21078862 PMC3075755

[B39] Lee K. Y. Park S.-J. Lee K.-A. Kim S.-H. Kim H. Meroz Y. (2018). Photosynthetic artificial organelles sustain and control ATP-dependent reactions in a protocellular system. Nat. Biotechnol. 36, 530–535. 10.1038/nbt.4140 29806849

[B40] Lozier R. H. Bogomolni R. A. Stoeckenius W. (1975). Bacteriorhodopsin: A light-driven proton pump in *Halobacterium halobium* . Biophys. J. 15, 955–962. 10.1016/s0006-3495(75)85875-9 1182271 PMC1334761

[B41] Lussier F. Staufer O. Platzman I. Spatz J. P. (2021). Can bottom-up synthetic biology generate advanced drug-delivery systems? Trends Biotechnol. 39, 445–459. 10.1016/j.tibtech.2020.08.002 32912650

[B42] Miller T. E. Beneyton T. Schwander T. Diehl C. Girault M. Mclean R. (2020). Light-powered CO_2_ fixation in a chloroplast mimic with natural and synthetic parts. Science 368 (6491), 649–654. 10.1126/science.aaz6802 32381722 PMC7610767

[B43] Noble J. M. Lubieniecki J. Savitzky B. H. Plitzko J. Engelhardt H. Baumeister W. (2018). Connectivity of centermost chromatophores in *Rhodobacter sphaeroides* bacteria. Mol. Microbiol. 109, 812–825. 10.1111/mmi.14077 29995992

[B44] Noireaux V. Libchaber A. (2004). A vesicle bioreactor as a step toward an artificial cell assembly. Proc. Natl. Acad. Sci. U. S. A. 101, 17669–17674. 10.1073/pnas.0408236101 15591347 PMC539773

[B45] Otrin L. Kleineberg C. da Silva L. C. Landfester K. Ivanov I. Wang M. H. (2019). Artificial organelles for energy regeneration. Adv. Biosyst. 3, 1800323. 10.1002/adbi.201800323 32648709

[B46] Pitard B. Richard P. Duñarach M. Girault G. Rigaud J.-L. (1996). ATP synthesis by the F_0_F_1_ ATP synthase from thermophilic *Bacillus* PS3 reconstituted into liposomes with bacteriorhodopsin. Eur. J. Biochem. 235, 769–778. 10.1111/j.1432-1033.1996.00769.x 8654428

[B47] Racker E. Stoeckenius W. (1974). Reconstitution of purple membrane vesicles catalyzing light-driven proton pump and adenosine triphosphate Formation. J. Biol. Chem. 249, 662–663. 4272126

[B48] Rampioni G. Mavelli F. Damiano L. D'Angelo F. Messina M. Leoni L. (2014). A synthetic biology approach to bio-chem-ICT: First moves towards chemical communication between synthetic and natural cells. Nat. Comput. 13, 333–349. 10.1007/s11047-014-9425-x

[B49] Richard P. Graber P. (1992). Kinetics of ATP synthesis catalyzed by the H^+^-ATPase from chloroplasts (CF_0_F_1_) reconstituted into liposomes and coreconstituted with bacteriorhodopsin. Eur. J. Biochem. 210, 287–291. 10.1111/j.1432-1033.1992.tb17419.x 1446676

[B50] Rigaud J. L. Pitard B. Levy D. (1995). Reconstitution of membrane proteins into liposomes: Application to energy-transducing membrane proteins. Biochimica Biophysica Acta 1231, 223–246. 10.1016/0005-2728(95)00091-v 7578213

[B51] Sato W. Zajkowski T. Moser F. Adamala K. P. (2022). Synthetic cells in biomedical applications. Nanomed Nanobiotechnol 14, e1761. 10.1002/wnan.1761 PMC8918002 34725945

[B52] Schäfer G. Engelhard M. Müller V. (1999). Bioenergetics of the archaea. Microbiol. Mol. Biol. Rev. 63 (3), 570–620. 10.1128/mmbr.63.3.570-620.1999 10477309 PMC103747

[B53] Schoonen L. van Hest J. C. M. (2016). Compartmentalization approaches in soft matter science: From nanoreactor development to organelle mimics. Adv. Mater. 28 (6), 1109–1128. 10.1002/adma.201502389 26509964

[B54] Schrodinger E. (1945). What is life the physical aspect of the living cell and mind and matter. Cambridge Cambridge University Press.

[B55] Schwille P. Spatz J. Landfester K. Bodenschatz E. Herminghaus S. Sourjik V. (2018). MaxSynBio: Avenues towards creating cells from the bottom up. Angew. Chem. Int. Ed. 57, 13382–13392. 10.1002/anie.201802288 29749673

[B56] Sener M. Strümpfer J. Abhishek S. Hunter C. N. Schulten K. (2016). Overall energy conversion efficiency of a photosynthetic vesicle. eLife 5, e09541. 10.7554/elife.09541 27564854 PMC5001839

[B57] Sener M. Strümpfer J. Timney J. A. Freiberg A. Hunter N. A. Shulten K. (2010). Photosynthetic vesicle architecture and constraints on efficient energy harvesting. Biophys. J. 99, 67–75. 10.1016/j.bpj.2010.04.013 20655834 PMC2895385

[B58] Shin K. (2019). Artificial cells containing sustainable energy conversion engines. Sciences 3, 573–578. 10.1042/ETLS20190103 33523178

[B59] Shirt-Ediss B. Ruiz-Mirazo K. Mavelli F. Solé R. V. (2014). Modelling lipid competition dynamics in heterogeneous protocell populations. Sci. Rep. 4, 5675. 10.1038/srep05675 25024020 PMC4097352

[B60] Staehelin A. L. Paolillo D. J. (2020). A brief history of how microscopic studies led to the elucidation of the 3D architecture and macromolecular organization of higher plant thylakoids. Photosynth. Res. 145, 237–258. 10.1007/s11120-020-00782-3 33017036 PMC7541383

[B61] Stano P. (2022). A four-track perspective for bottom-up synthetic cells. Front. Bioeng. Biotechnol. 10, 1029446. 10.3389/fbioe.2022.1029446 36246382 PMC9563707

[B62] Staufer O. De Lora J. A. Bailoni E. Bazrafshan A. Benk A. S. Jahnke K. (2021). Building a community to engineer synthetic cells and organelles from the bottom-up. eLife 10, e73556. 10.7554/eLife.73556 34927583 PMC8716100

[B63] Steinberg-Yfrach G. Liddell P. A. Hung S.-C. Moore A. L. Gust D. Moore T. A. (1997). Conversion of light energy to proton potential in liposomes by artificial photosynthetic reaction centres. Nature 385, 239–241. 10.1038/385239a0

[B64] Steinberg-Yfrach G. Rigaud J.-L. Durantini E. N. Moore A. L. Gust D. Moore T. A. (1998). Light-driven production of ATP catalysed by F0F1-ATP synthase in an artificial photosynthetic membrane. Nature 392, 479–482. 10.1038/33116 9548252

[B65] Toparlak O. D. Mansy S. S. (2019). Progress in synthesizing protocells. Exp. Biol. Med. 244, 304–313. 10.1177/1535370218816657 PMC6435886 30509137

[B66] Van Der Bend R. L. Cornelissen J. B. W. J. Berden J. A. Van Dam K. (1984). Factors defining the functional coupling of bacteriorhodopsin and ATP synthase in liposomes. Biophys. Acta 767, 87–101. 10.1016/0005-2728(84)90082-3

[B67] van Dijck P. W. M. van Dam K. (1982). Bacteriorhodopsin in phospholipid vesicles. Methods Enzymol. 88, 17–25.

[B68] Wagner N. Gutweiler M. Pabst R. Dose K. (1987). Coreconstitution of bacterial ATP synthase with monomeric bacteriorhodopsin into liposomes. A comparison between the efficiency of monomeric bacteriorhodopsin and purple membrane patches in coreconstitution experiments. Eur. J. Biochem. 165, 177–183. 10.1111/j.1432-1033.1987.tb11209.x 2883008

[B69] Walde P. Cosentino K. Engel H. Stano P. (2010). Giant vesicles: Preparations and applications. ChemBioChem 11, 848–865. 10.1002/cbic.201000010 20336703

[B70] Whitmarsh J. (1999). “The photosynthetic process,” in Concepts in photobiology: Photosynthesis and photomophogenesis. Editors Renger G. Sopory S. K. Irrgang K. D. (Dordrecht: Springer G. S.).

[B71] Woese C. R. (1987). Bacterial evolution. Microbiol. Rev. 51, 221–271. 10.1128/mr.51.2.221-271.1987 2439888 PMC373105

[B72] Woese C. R. Stackebrandt E. Weisburg W. G. Paster B. J. Madigan M. T. Fowler V. J. (1984). The phylogeny of purple bacteria: The alpha subdivision. Syst. Appl. Microbiol. 5 (3), 315–326. 10.1016/s0723-2020(84)80034-x 11541974

[B73] Woronowicz K. Niederman R. A. (2010). Proteomic analysis of the developing intracytoplasmic membrane in *Rhodobacter sphaeroides* during adaptation to low light intensity. New York: Springer. 10.1007/978-1-4419-1528-3_10 20532741

